# Evidence on antidepressant withdrawal: an appraisal and reanalysis of a recent systematic review

**DOI:** 10.1017/S0033291725100652

**Published:** 2025-07-22

**Authors:** Joanna Moncrieff, Harriet Hobday, Anders Sørensen, John Read, Martin Plöderl, Michael Hengartner, Caroline Kamp, Janus Jakobsen, Sophie Juul, James Davies, Mark Horowitz

**Affiliations:** 1 Division of Psychiatry, University College London, London, UK; 2 Research and Development Department, North East London NHS Foundation Trust (NELFT), Essex, UK; 3 Copenhagen Trial Unit, Centre for Clinical Intervention Research, The Capital Region, Copenhagen University Hospital – Rigshospitalet, Copenhagen, Denmark; 4 Department of Clinical Psychology, University of East London, London, UK; 5 University Clinic of Psychiatry Psychotherapy and Psychosomatics, Paracelsus Medical University Salzburg, Austria; 6 Department of Applied Psychology, Kalaidos University of Applied Sciences Zurich, Switzerland; 7 Department of Psychology, University of Copenhagen, Copenhagen, Denmark; 8 Department of Regional Health Research, The Faculty of Heath Sciences, University of Southern Denmark, Odense, Denmark; 9 Stolpegaard Psychotherapy Centre, Mental Health Services in the Capital Region of Denmark, Gentofte, Denmark; 10 Department of Medical Anthropology and Psychology, University of Roehampton, London, UK; 11 Institute of Pharmaceutical Sciences, King’s College London, London, UK

**Keywords:** antidepressant discontinuation, antidepressant withdrawal, meta-analysis, misclassification of withdrawal, spontaneous reporting

## Abstract

**Background:**

There has been debate about the frequency and severity of antidepressant withdrawal effects.

**Methods:**

We set out to appraise and reanalyze an influential systematic review by Henssler and colleagues that concluded that withdrawal effects are not particularly common and rarely severe. We repeated the meta-analysis, including only studies where data were derived from systematic measures of withdrawal symptoms.

**Results:**

Most data in the Henssler review are derived from pharmaceutical industry–sponsored efficacy studies in which withdrawal was a minor consideration. Shortcomings of the review include the use of spontaneously reported adverse events to estimate withdrawal symptoms, potential misclassification of withdrawal symptoms as relapse, inclusion of data from retrospective case-note studies, short duration of prior antidepressant use, short observation periods, the overlooking of differences between placebo and drug withdrawal effects, and the use of questionable proxies for severe withdrawal. There were also discrepancies and uncertainties in some figures used. In our reanalysis, we included only the five studies that used a systematic and relevant method to assess the incidence of any withdrawal symptom. Prior treatment was short-term (12 weeks or less) in all but one of these. The pooled percentage was 55% (95% confidence interval, CI, 31% to 81%; *N* = 601) without subtracting nocebo effects, with high heterogeneity.

**Conclusions:**

Henssler’s review is based on unreliable data and does not provide an adequate basis for the evaluation of antidepressant withdrawal effects. Further good-quality research on antidepressant withdrawal is required.

## Introduction

Antidepressants are widely prescribed, and their use is increasing across the globe (Alabaku et al., 2023). Almost a fifth of the population of the UK and the US use an antidepressant each year, and people frequently take them for long periods. Fifty per cent of users in the UK have taken them for more than a year and almost the same proportion in the US for more than five years (Brody & Gu, 2020; NHS Digital, 2023; Public Health England, 2019).

Initially, antidepressant withdrawal symptoms were generally thought to be mild and short-lived (Iacobucci, 2019; Sørensen, Jørgensen, & Munkholm, 2022a). However, a review published in 2019 suggested withdrawal symptoms occurred in 56% of participants across included studies and that nearly half of those reported the symptoms to be severe (Davies & Read, 2019a). Some guidelines were updated to reflect this evidence (Burn, Horowitz, Roycroft, & Taylor, 2020; Iacobucci, 2019; NICE, 2022b).

However, there was debate about the results of this review (Davies & Read, 2019b; Jauhar & Hayes, 2019). Subsequently, an influential review by Henssler et al. (2024) appeared to suggest that antidepressant withdrawal effects might be less common and only rarely severe.

On the basis of data from 62 cohorts from randomized trials and other studies, Henssler et al. estimated the incidence of ‘any’ withdrawal symptom to be 31% among those coming off an antidepressant. By deducting the incidence of symptoms following the discontinuation of placebo in an overlapping set of 22 trials, they concluded ‘the frequency of antidepressant discontinuation symptoms to be in the range of approximately 15%, thus affecting about one in six to one in seven patients’ (p. 534) (Henssler et al., 2024). They reported that only 3% of participants experienced severe withdrawal symptoms.

Henssler et al. claimed to have provided ‘a more comprehensive view’ than previous research (p. 534)(Henssler et al., 2024).

Since this review was published, another systematic review produced a pooled incidence rate of 43% in mostly short-term trials (Zhang et al., 2024). To shed light on the seeming discrepancies between reviews and to inform clinicians and patients about the current evidence, we set out to appraise the Henssler et al.’s review, including the nature and quality of the data it included. As a secondary aim, we planned a meta-analysis of the occurrence of any withdrawal symptom in studies included by Henssler et al. that had applied a systematic and relevant assessment of withdrawal symptoms.

## Methods

We inspected the original publications on the 62 study cohorts (some studies involved more than one cohort) included in Henssler et al.’s incidence analysis and the 19 studies included in the analysis of severe withdrawal. We extracted data on details not reported in the original review, including the method of assessment of withdrawal symptoms used in the analysis, conflicts of interest and sponsorship, and the potential for the misclassification of withdrawal symptoms as relapse or deterioration of the underlying condition. We also re-extracted data on the occurrence of withdrawal as defined by Henssler et al. (at least one withdrawal symptom). All data extraction was double-checked.

The possibility of misclassification was evaluated after inspecting the included studies according to the following criteria: ‘high potential’ for misclassification where data used to evaluate withdrawal were collected non-systematically and concurrently with measures of psychiatric symptoms or relapse, and there was evidence suggestive of misclassification (such as the non-inclusion of typical emotional symptoms among reported effects of withdrawal); ‘medium potential’ where misclassification may have influenced ratings of withdrawal; and ‘low potential’ where misclassification was judged not likely to have been a significant problem. Further details about the basis of these judgments are provided in the Supplementary Table.

We reanalyzed studies included in the Henssler et al.’s review according to a predefined analysis plan (see Supplementary material). We included studies that had usable data derived from a systematic and relevant method of assessment of withdrawal symptoms. We defined this as the use of a structured questionnaire or method that captured common withdrawal symptoms. We excluded studies that used measures designed for other purposes that did not cover withdrawal symptoms. Henssler et al. kindly supplied clarifications of the origin of their figures in certain instances. As in the original review, we analyzed the proportion of people who entered the study who reported at least one withdrawal symptom and conducted the meta-analysis of proportions, using the Logit method based on the inverse variance. We used R’s ‘meta’ package. Details of the code are available at the Open Science Foundation (OSF) https://osf.io/de3gj.

## Results

In examining Henssler’s review, we identified several strengths, including the use of systematic searches, risk of bias assessments, the evaluation of withdrawal symptoms in people withdrawn from placebo, and the exploration of potential predictors of withdrawal. However, we also identified some significant limitations, many of which were not readily apparent in the published paper. These include the use of spontaneously reported adverse events to estimate withdrawal symptoms (including in many studies that also used a structured instrument), potential misclassification of withdrawal symptoms as relapse, inclusion of data from retrospective case-note studies, short duration of prior antidepressant use in many studies, short observation periods, lack of consideration of differences between placebo and drug withdrawal effects, and the use of questionable proxies for severe withdrawal. There were also some significant discrepancies and uncertainties in the figures used.

## Design of studies

The majority of the 62 studies or cohorts included in the incidence analysis were acute efficacy studies, extension studies, or relapse prevention studies, in which withdrawal effects were an incidental concern and not reliably measured (see below). Only 16 (26%) were designed primarily to study withdrawal, and these were mostly small (See Table 1).

**Table 1.** Characteristics of studies used in Henssler et al.’s analysis of the incidence of any withdrawal symptom (eFigure 1 in Henssler’s supplementary appendix)

Study	Assessment of withdrawal a primary or secondary aim of the study?^a^	Study design	Measure of withdrawal symptoms used in Henssler et al’s incidence analysis	Condition	Duration of prior use	Observation period (after the end of treatment including tapering)	Funding and conflicts of Interest (COI)	Henssler et al’s incidence figures	Discrepancies with incidence figures	Potential misclassification of withdrawal^b^
Allgulander et al. (2006)	Secondary	A randomized, placebo-controlled discontinuation trial of escitalopram for relapse prevention following 12 weeks of OL treatment.	Adverse events in the 2 weeks after randomization in the placebo group and the final taper period	GAD	12 weeks (N = 188). Up to 88 weeks (N = 116)	1–2 weeks	Drug company funded	96/304		High
Bainum (2017)	Primary	Retrospective case notes review among people admitted to ITU who stopped an antidepressant (SSRI or SNRI)	Authors’ list of symptoms	Mixed	not specified	72 hours	A COI statement reports no COIs	18/41		Low
Bakish et al. (2014)^c^	Secondary	An 8-week placebo-controlled efficacy trial of levomilnacipran followed by a down-taper period.	Adverse events in taper-down period	Depression	8 weeks	30 days	Drug company funded	15/376		Medium
Baldwin (2006)	Secondary	A 12-week placebo-controlled efficacy trial of escitalopram and paroxetine with a subsequent 2-week double blind taper-down period	Adverse events in taper-down period (DESS also used – mean scores reported only)	GAD	12 weeks	2 weeks	Drug company funded	111/459		Medium
Bhuamik (1996)	Primary	Retrospective case notes review of people who discontinued fluoxetine or paroxetine	Not specified.	Depression in people with learning disability	Mean 8 months for fluoxetine; 7.5 months for paroxetine	not specified	No COI or funding statement	5/12		Medium
Black et al. (1993)	Secondary	Uncontrolled study of people withdrawn abruptly from fluvoxamine	Spontaneous reports	Panic disorder	7–8 months	14 days	Drug company funded	12/14		Low
Bourgeois (1991)	Secondary	Uncontrolled, 6-week study of tianeptine with withdrawal evaluated after discontinuation	‘clinical signs and symptoms’	Depression with melancholic features	6 weeks	1 week	Probable drug company funding	0/14	Denominator refers to observations. It should be 30 (number of participants).	Medium
Ceccherini-Nelli (1993)	Primary	Uncontrolled study of people withdrawn from tricyclic antidepressants.	Open-ended questions	9 participants had depression, 1 had schizophrenia	Not stated	Not stated	No COI or funding statement	7/10		Low
Charney (1982)	Primary	Uncontrolled study of withdrawal of tricyclic antidepressants and placebo substitution	Nurses’ psychological symptom ratings.	Depression (including bipolar depression)	5 weeks for 5 participants, 6 weeks for one, and one not specified	10–21 days	US State funding, no COI statement	2/7		Low
Clauw (2013)	Secondary	A 12-week, placebo-controlled discontinuation (relapse prevention) trial of milnacipran following long-term OL treatment.	Adverse events in the placebo group during the whole course of the trial	Fibromyalgia	Mean 36 months (17.9–54.4)	12 weeks	Drug company funded	29/50		Medium
Cohen et al., (2004)	Secondary	Uncontrolled study to evaluate efficacy and tolerability of intermittent venlafaxine.	Any DESS symptom 2–5 days after discontinuation	Premenstrual syndrome	30 days in two cycles (15 days and 15 days with a gap of 2 weeks)	2–5 days	Drug company funded	8/11		Low
Coupland (1996)	Primary	Retrospective case notes review of patients who had stopped an SSRI or clomipramine	Clinician report	Mostly anxiety and ‘mood disorders’	mean between 12 and 37 weeks	2 weeks (paroxetine, fluvoxamine, and clomipramine). More than 4 weeks (fluoxetine and sertraline).	No COI or funding statement	31/171	Withdrawal events should be 21, not 31	Medium
Durgam (2019)	Secondary	A 26-week, placebo-controlled discontinuation (relapse prevention) trial of milnacipran following 20 weeks of OL treatment, followed by a ‘down taper’ phase.	Adverse events in the placebo group during the course of the trial	Depression	20 weeks	Up to 26 weeks	Drug company funded	82/159		High
Fava (1997)	Secondary	An 8-week, placebo-controlled acute study of extended-release venlafaxine	‘open-ended question’ 5 days after discontinuation	Depression	8 weeks	mean 5 days	Drug company funded	7/9		Medium
Favaro (2001)	Primary	Retrospective case notes review after use and discontinuation of sertraline	‘At least two symptoms typical of SSRI-withdrawal’. No further details	Anorexia nervosa	25.5 weeks	7 days	No COI or funding statement	6/24	Figures not consistent with criteria (one symptom)	Medium
Feiger (1999)	Secondary	A placebo-controlled, discontinuation trial of nefazadone for relapse prevention following 16 weeks of OL treatment.	Adverse events in the first two weeks after randomization to placebo	Depression	16 weeks	not specified	Drug company funded	15/66		High
Ferguson (2012)	Secondary	Uncontrolled study of ‘safety and efficacy’ of up to one year treatment with desvenlafaxine, followed by a taper over 1–2 weeks	Adverse events	Depression	51.8–52.8 weeks	7 days post-discontinuation	Drug company funded	54/104	Denominator unreliable due to omission or modification of the taper in some participants	Medium
Gallagher (2012)	Primary	A 2-week taper phase comparing different methods of tapering following a 15-week OL trial of desvenlafaxine.	Adverse events (DESS also used – mean scores reported only)	Vaso-motor symptoms of the menopause	15–16 weeks	2–4 weeks	Drug company funded	89/384		Medium
GlaxoSmithKline (1992)	Primary	Abrupt discontinuation of imipramine or paroxetine and single-blind placebo substitution for 2 weeks following 6–12 weeks of OL treatment	Adverse events	Depression	6–12 weeks	10–14 days	Drug company funded and conducted	71/186		Medium
Higuchi (2016)	Secondary	An 8-week, placebo-controlled trial of venlafaxine extended release followed by a 2-week taper period and a 2-week follow-up	Adverse events	Depression	8 weeks	2 weeks	Drug company funded	99/354	Denominator should be 307, not 354	Medium
Ivgy-May (2015)	Secondary	A two-week, placebo-controlled trial of three different doses of esmirtazapine for insomnia, followed by a 7-day post-discontinuation follow-up period	Adverse events	Primary insomnia.	2 weeks	1 week	Drug company funded	4/390	Denominator unreliable due to likely dropouts/loss to follow-up.	Low
Jain (2012)	Secondary	A 6-week placebo-controlled, acute efficacy study of vortioxetine followed by a 2-week post-discontinuation follow-up	Adverse events	Depression	6 weeks	2 weeks	Drug company funded	25/300	Denominator unreliable due to likely dropouts/loss to follow-up.	Medium
Kamijima (2005)	Secondary	A placebo-controlled, discontinuation study of sertraline for relapse prevention after 8 weeks of OL treatment.	Adverse events in the placebo group during the whole course of the trial	Panic disorder	8 weeks	8 weeks	Drug company funded	42/121	Events should be 40	Medium
Khan et al. (2014)	Primary	Double-blind, 4-week comparison of abrupt discontinuation of desvenlafaxine, a 1-week taper, and continuation treatment after 24-week OL treatment	Adverse events (DESS also used – mean scores reported only)	Depression	24 weeks	1–3 weeks post discontinuation	Drug company funded	129/285		Medium
Koran (2003)	Secondary	A 9-week placebo-controlled discontinuation trial of citalopram following a 7-week OL phase.	Adverse events in the placebo group during the whole course of the trial	Compulsive shopping disorder	7 weeks	9 weeks	Drug company funded	2/ 8		High
Kornstein et al. (2006)	Secondary	Placebo-controlled discontinuation trial of escitalopram for relapse prevention following 16 weeks of OL treatment and 8 weeks of treatment with another drug.	Adverse events in the placebo group in the first 2 weeks after randomization	Depression	24 weeks	2 weeks	Drug company funded	27/66		High
Kragh-Sorensen et al. (1974)	Secondary	Abrupt discontinuation of nortriptyline after at least 20 weeks of treatment and substitution with placebo for one week.	An 11-item checklist (Asberg, 1970) was used, although results were not reported	Depression	at least 20 weeks	1 week	Non-drug company funding declared. No COI statement.	2/10		High
Kramer et al. (1961)	Primary	A retrospective case notes review of patients who stopped imipramine	Not specified.	Not reported	19 patients had used imipramine for < 2 months, 26 for > 2 months or more	up to 48 hours	Drug company ‘cooperation’	25/45		Medium
Liebowitz (2009)	Secondary	Randomized placebo-controlled trial of venlafaxine ER followed by a 2-week taper phase and 4–10 day follow-up	Adverse events	Panic disorder	up to 10 weeks	4–10 days	Drug company funded	70/163	Denominator unreliable due to modification or omission of taper and dropouts/loss to follow-up.	Medium
Mago (2013)	Secondary	An OL extension study of up to 48 weeks of milnacipran (subsequent to three placebo-controlled trials), followed by a taper-down and follow-up period of up to 4 weeks.	Adverse events	Depression	median 40 weeks	1–2 weeks post discontinuation	Drug company funded	75/490		Medium
Mallya (1993)	Primary	Retrospective case notes review of participants who discontinued fluvoxamine following a placebo-controlled trial and one-year OL extension phase	Hopkins symptom checklist	OCD	52 weeks	not clear	No COI or funding statement	4/17		Medium
Mease (2010)	Secondary	A pooled analysis of two 26-week, OL extension trials of duloxetine at various doses (preceded by two 26-week placebo-controlled trials) with a two-week taper and follow-up phase	Adverse events	Fibromyalgia with or without depression	Between 26 and 52 weeks	1 week	Drug company funded	29/122	Denominator unreliable due to dropouts/loss to follow-up.	Medium
Montgomery (2009) (flexible), the figures given are actually for all 9 short-term studies – fixed and flexible dose	Secondary	Henssler’s figures refer to 9 fixed and flexible-dose, short-term, placebo-controlled trials of desvenlafaxine, which were followed by a taper period of between 0 and 2 weeks and a follow-up of between 1 and 3 additional weeks	Adverse events (DESS also used- mean scores reported only).	Depression	8 weeks	1–3 weeks	Drug company funded	455/1141		Medium
Montgomery (2009) (fixed)	Secondary	The figures are for the fixed-dose studies included in the Montgomery 2009 ‘flexible’ dose studies	Adverse events (DESS also used- mean scores reported only).	Depression	8 weeks	1–3 weeks	Drug company funded	409/947	Double counted (included in Montgomery 2009 ‘flexible dose’)	Medium
Montgomery (2005)	Secondary	A placebo-controlled discontinuation study of escitalopram for relapse prevention after 12 weeks of open-label treatment.	Adverse events in the placebo group two weeks after randomization. (DESS also used – mean scores reported only).	Generalized social anxiety disorder	12 weeks	2 weeks	Drug company funded	101/181		High
Montgomery (2013)	Secondary	A 10-week, placebo-controlled efficacy trial of milnacipran followed by a 1-week taper period and 1 week follow-up	Adverse events	Depression	10 weeks	1 week	Drug company funded	24/278	Denominator unreliable due to dropouts/loss to follow-up.	Medium
Mourad et al. (1998)	Primary	An uncontrolled study of withdrawal of mixed antidepressants (tricyclic antidepressants, SSRIs, MAOIs, and trazadone)	A benzodiazepine withdrawal symptoms scale with two added questions	Mixed diagnoses	15 days or more	3 days	No COI or funding statement	14/16		Low
Murata et al. (2010)^c^	Primary	An uncontrolled study of genes associated with paroxetine withdrawal in the 7 days following discontinuation or reduction.	A list of withdrawal symptoms	Depression, anxiety, and pain	106 weeks	1 week	Non-drug company funding declared. COI statement reports no COIs.	20/56		Low
Oehrberg (1995)	Secondary	A 12-week placebo-controlled efficacy trial of paroxetine, followed by abrupt discontinuation and placebo substitution for 2 weeks	Adverse events	Panic disorder	12 weeks	2 weeks	Drug company funded	19/55		Medium
Otani (1991)	Primary	Uncontrolled study of mianserin withdrawn abruptly or over 1 month, with a two-week follow-up	UKU side effects scale	Mostly depression	mean 22 weeks	2 weeks	No COI or funding statement.	1/22		Medium
Perahia (2009)	Secondary	A 52-week placebo-controlled discontinuation trial of duloxetine for relapse prevention (preceded by 28–34 weeks of OL treatment) followed by a down-taper and follow-up of 2–3 weeks.	Adverse events during taper and follow-up	Depression	80–86 weeks	The taper phase and follow-up lasted 2–3 weeks concurrently	Drug company funded	14/61		Medium
Perahia (2005) acute studies	Secondary	Pooled data from 6 placebo-controlled efficacy trials of duloxetine lasting 8–9 weeks that were followed by abrupt discontinuation and a follow-up of 1–2 weeks	Adverse events	Depression	8–9 weeks	1–2 weeks	Drug company funded	217/490		Medium
Perahia (2005) extension studies	Secondary	Pooled analysis of two, 26-week OL extension studies (following 8-week, placebo-controlled trials), followed by abrupt discontinuation and follow-up of 1–2 weeks	Adverse events	Depression	34 weeks	1–2 weeks	Drug company funded	22/242		Medium
Rapaport (2001)	Secondary	A 28-week, placebo-controlled, discontinuation trial of sertraline for relapse prevention following a 52-week OL phase (which followed a 10-week efficacy trial).	Adverse events in the placebo group during the whole course of the discontinuation trial	Panic disorder	52 weeks (plus 10 weeks for those who were on the active drug in the initial efficacy trial)	28 weeks	Drug company funded	9/89	Incorrect figures. 9 refers to the number who withdrew due to an adverse event (the number who experienced any adverse event is not reported).	High
Raskin (2003) (results and details reported in Perahia, 2005)	Secondary	Uncontrolled study of duloxetine at different doses, followed by abrupt discontinuation and a 2-week follow-up phase	Adverse events	Depression	52 weeks	2 weeks	Drug company funded	281/553		Medium
Ravindran (2007)	Secondary	Open study of citalopram for premenstrual syndrome taken from onset of symptoms to start of menses for 2 cycles	Adverse events	Premenstrual syndrome	mean 11.6 days (over 2 menstrual cycles)	not specified	Author COIs but not funded	0/7		Low
Rickels (2010) open-label study	Secondary	12-week OL study of venlafaxine followed by a two-week taper period, including those who did not enter the subsequent relapse prevention trial.	Adverse events	Depression	12 weeks	2-week taper period (not clear if there is any post-discontinuation follow-up)	Drug company funded	68/218	Denominator unreliable due to modification or omission of the taper	Medium
Rickels (2010) Relapse prevention	Secondary	A 24-week, placebo-controlled discontinuation trial of venlafaxine for relapse-prevention following a 12-week OL treatment phase, followed by a 1–2 week taper phase.	Adverse events in the taper phase (DESS also used – mean scores reported only)	Depression	36 weeks	2-week taper period (not clear if there is any post-discontinuation follow-up)	Drug company funded	101/190	Denominator unreliable due to modification or omission of the taper	Medium
Rosenthal et al. (2013)	Secondary	A 6-month, placebo-controlled discontinuation trial of desvenlafaxine for relapse prevention (following a 20-week OL phase) followed by a 1-week taper and 1-week follow-up.	Adverse events after the end of OL treatment for those who did not enter the trial, and during the taper and follow-up phase at the end of the double-blind trial for those who did	Depression	46 weeks	1 week	Drug company funded	53/300	Denominator unreliable due to dropouts/loss to follow-up.	Medium
Santonastaso (2001)	Secondary	An uncontrolled, 14-week study of sertraline followed by discontinuation	Not specified	Anorexia nervosa	14 weeks	Not specified	No funding or COI statement	2/7		Medium
Saxe (2012)	Secondary	A 12-week placebo-controlled trial of milnacipran followed by a 2-week, placebo-controlled, discontinuation trial to evaluate ‘loss of efficacy’.	Adverse events	Fibromyalgia	12 weeks	2 weeks	Drug company funded	29/178		High
Stein et al. (1996)	Secondary	An 11-week, OL trial of paroxetine followed by a randomized, placebo-controlled relapse prevention trial.	Not specified	Social phobia	11 weeks	Not specified	Not drug company funded. No COI statement.	2/8		High
Stein et al. (2008)	Secondary	A 12-week, placebo-controlled trial of agomelatine. Withdrawal assessed 1 week after the end of treatment.	Any DESS symptom	GAD	12 weeks	1 week	Drug company funded	25/63		Low
Steiner (2005)	Secondary	A placebo-controlled trial of paroxetine during the luteal phase (14 days) for pre-menstrual dysphoric disorder.	Adverse events measured 3 days after start of menses	Pre-menstrual dysphoric disorder	14 days	3 days	Probable drug-company funding	46/246		Low
Tourian et al. (2011)	Secondary	An open-label extension study of up to 10 months (following six 8-week efficacy trials of desvenlafaxine), followed by a 7-day taper period	Adverse events	Depression	up to 12 months	1 week	Drug company funded	584/1395	Denominator incorrect and unreliable due to dropouts/loss to follow-up and modification or omission of taper.	Medium
Tourian et al. (2009)	Secondary	An 8-week, placebo-controlled trial of desvenlafaxine and duloxetine followed by a 7-day taper period	Adverse events elicited by ‘specific questions’ about withdrawal symptoms (DESS also used – mean scores reported only)	Depression	8 weeks	7 days	Drug company funded	240/455		Low
Tyrer (1984)	Primary	Uncontrolled withdrawal study of tricyclic antidepressants and phenelzine	Spontaneously reported new symptoms during withdrawal	Mixed anxiety and depression	mean 10 to 16 months	4 weeks	No funding or COI statement	16/51		High
Vandel (2004)	Secondary^a^	A randomized comparative trial of milnacipran and paroxetine with discontinuation after 6 weeks for some and a further 18 weeks for others	Adverse events	Depression	6 weeks (N = 90) and 24 weeks (N = 53)	1 week	Drug company funded	36/143		Medium
Wade (2007)	Secondary	A comparative trial of escitalopram and duloxetine followed by a 2-week taper period	Adverse events	Depression	24 weeks	4 weeks	Drug company funded	77/226		Medium
Yasui-Furukori (2016)	Primary	Uncontrolled study of withdrawal of escitalopram	3 or more DESS symptoms	Depression	> 6 months	4 weeks	Author COIs but not funded	14/25	Figures refer to participants who had 3 or more DESS symptoms (i.e. do not fit Henssler’s specified criteria)	Low
Zajecka (1998a)	Secondary	Placebo-controlled, 6-week, discontinuation trial of fluoxetine for ‘maintenance treatment’ after 12 weeks of OL treatment.	Adverse events in the placebo group 6 weeks after randomization	Depression	12 weeks	6 weeks	Probable drug company funding	23/58	Non-optimal figures relating to adverse events at 6 weeks. Figures for an adverse event over the course of the 6 weeks were 64/96 (67%) in the placebo (discontinued) group	Medium
Zajecka (1998b) (conference abstract)	Secondary	Two placebo-controlled discontinuation trials of nefazadone for ‘maintenance treatment’.	Adverse events in the placebo groups 14 days post-randomization	Depression	Not specified	14 days	Probable drug company funding	27/130		Medium

Abbreviations: GAD, generalized anxiety disorder; OCD, obsessive compulsive disorder; SSRI, selective serotonin reuptake inhibitor; SNRI, serotonin and noradrenaline reuptake inhibitor; OL, Open Label; DESS, Discontinuation-Emergent Signs and Symptoms.

a We have classified studies according to the aim of the original study from which the data were gathered. Some papers focus on data on withdrawal from studies that were set up with a different aim, hence we have classified them as ‘secondary’ whereas Henssler et al. classified them as ‘primary’.

b ‘High’ = high potential for misclassification because withdrawal and relapse/psychiatric symptoms measured concurrently with evidence suggestive of misclassification (such as the noninclusion of typical emotional symptoms among reported effects of withdrawal); ‘Medium’ = medium potential for misclassification where misclassification may have influenced ratings of withdrawal; ‘Low’ = misclassification not likely to have been a significant problem. See Supplementary Table S1 for more detailed rationale for individual studies.

c Some or all participants did not stop their antidepressant.

Forty-six (74%) of the 62 studies had definite or probable funding from a pharmaceutical company (Table 1). Since funded studies were larger than non-funded studies, they accounted for 96.2% (12,119/12,603) of the participants included in the analysis.

## Assessment of withdrawal effects

A fundamental problem with the review is the manner of assessment of withdrawal symptoms (Table 1). In 52 of the 62 study cohorts, figures were derived from data on adverse events, responses to open questions, clinician judgment, or no method was specified. Where details were provided, adverse events and symptoms were ‘spontaneously reported’ in all but one study. In this study, specific withdrawal symptoms were enquired about during the measurement of adverse events (Tourian et al., 2009). Whether adverse events counted as withdrawal effects was further determined by the subjective judgment of the researchers who decided ‘if they occurred for the first time or worsened following discontinuation of treatment ’(p. 208) (Perahia, Kajdasz, Desaiah, & Haddad, 2005).

It is known that the detection of adverse events in studies designed to evaluate efficacy is unreliable, inconsistent, and likely to underestimate effects (Chrysant, 2008; Hammad, Pinheiro, & Neyarapally, 2011; Phillips, Hazell, Sauzet, & Cornelius, 2019). Ratings show poor reliability even for physical symptoms (Forster, Taljaard, Bennett, & van Walraven, 2012) and when raters are guided by a list of specific symptoms (Atkinson et al., 2012). In a trial of a chiropractic intervention, 88 times more adverse events were identified using proactive monitoring than when relying on spontaneous reports (Pohlman et al., 2020). Subjective adverse effects, including symptoms such as fatigue and emotional changes, are more likely to be under-detected than objective physical signs such as oedema (Chrysant, 2008). The reporting, as well as the detection of adverse effects in such studies, is also unreliable (Mayo-Wilson et al., 2019; Phillips et al., 2019).

Underestimation of adverse effects following antidepressant withdrawal is particularly likely because the most common symptoms include anxiety, fatigue, impaired concentration, and worsened mood, as documented in a study of over 1000 participants (Moncrieff, Read, & Horowitz, 2024), which overlap with symptoms of the disorders for which antidepressants are most commonly prescribed. Therefore, withdrawal symptoms can be overlooked or misclassified as symptoms of the underlying condition.

Eleven studies that rated withdrawal symptoms and mental disorder symptoms concurrently were rated as showing a ‘high potential’ for misclassification (See Table 1 and Supplementary Table S1).

In several of these studies, the authors acknowledged the problem by conducting sensitivity analyses of their efficacy measure, excluding data from the first few weeks after randomization (Allgulander, Florea, & Huusom, 2006; Kornstein et al., 2006; Rosenthal et al., 2013). However, they did not consider how the potential misclassification might have impacted the detection of withdrawal symptoms.

Only 13 of the 62 studies were rated as showing a low potential for misclassification.

In addition to these problems, five studies in the incidence analysis were retrospective case note reviews identifying reports of withdrawal symptoms entered by clinicians during routine clinical care (Table 1). Such studies are likely to miss all but the most distinctive and severe symptoms of withdrawal due to the lack of awareness of the range of effects (Guy et al., 2020).

## Use of structured instruments

Although 18 of the 62 studies included in Henssler’s incidence analysis used a structured instrument to assess withdrawal symptoms, in 10 of these, Henssler et al.’s analysis was based on adverse events because data from the instrument were not available in the required form (Table 2). In three studies, the instrument was developed for other purposes and did not include common antidepressant withdrawal symptoms. In one, data did not reflect the proportion of people experiencing ‘any’ symptom as per Henssler et al.’s criteria (Table 2) (Yasui-Furukori et al., 2016). In only four studies were withdrawal symptoms measured using a relevant instrument and reported in such a way as to be eligible for Henssler et al.’s analysis. Two of these used the Discontinuation-Emergent Signs and Symptoms (DESS) (Cohen et al., 2004; Stein et al., 1996) and two used similar instruments or sets of questions (Mourad, Lejoyeux, & Adès, 1998; Murata et al., 2010). One further study presented data on ‘specific’ adverse events that were elicited alongside the DESS questionnaire (Tourian et al., 2009).

**Table 2.** Studies included in Henssler et al.’s incidence analysis that used a structured instrument

Study	Measures used in study	Measure used in Henssler’s incidence calculation	Incidence rate (from Henssler et al) (%)
Baldwin (2006)	DESS and AEs	AEs	111/459 (24.2%)
Cohen et al. (2004)^a^	DESS	Any symptom on the DESS	8/11 (72.7%)
Gallagher (2002)	DESS and AEs	AEs	89/384 (23.2%)
Khan et al. (2014)	DESS and AEs	AEs	129/285 (45.3%)
Kragh-Sorensen et al. (1974)	11-item checklist (Asberg, 1970) (a checklist for side effects of tricyclic antidepressants. Does not cover common antidepressant withdrawal symptoms, including emotional and cognitive effects)	The statement that two patients had mild headaches. Checklist data are not presented	2/10 (20%)
Mallya (1993)	Hopkins checklist (a screening checklist for anxiety and depression- does not cover many common antidepressant withdrawal symptoms) retrospectively applied to medical notes	Any symptom on the Hopkins checklist	4/17 (23.5%)
Montgomery (2009) (pooled short-term studies. ‘Flexible’ studies according to Henssler)	DESS and AEs	AEs	455/1141 (39.9%)
Montgomery (2009) (Flexible or long-term)	DESS and AEs	AEs	Included in Montgomery (2009) pooled short-term studies
Montgomery (2005)	DESS and AEs	AEs	101/181 (55.8%)
Mourad (1998)^a^	A benzodiazepine withdrawal scale	Any symptom on the benzodiazepine withdrawal scale	14/16 (87.5%)
Murata et al. (2010)^a^	A scale similar to the DESS	Any symptom on the scale	20/56 (35.7%)
Otani (1991)	UKU side effects scale (a general drug side effects scale, developed in the 1980s, mainly focused on antipsychotic side effects. Many common antidepressant withdrawal symptoms are not covered)	Any symptom on the UKU side effects scale	1/22 (4.6%)
Rickels (2010) (end of open-label period)	DESS and AEs	AEs	68/218 (31.2%)
Rickels (2010) (end of double-blind)	AEs only (DESS was used in a different part of the study for those who continued into the double-blind period.)	AEs	101/190 (53.2%)
Stein et al. (2008)^a^	DESS	Any symptom on the DESS	25/63 (39.7%)
Tourian et al. (2009)^a^	DESS and AEs	‘specific’ adverse events	240/455 (52.7%)
Tyrer (1984)	Spontaneously reported ‘new symptoms’ during withdrawal were the basis of reported withdrawal symptoms. Separately, pre-specified criteria were used to attempt to distinguish increases in anxiety and depression scores due to withdrawal symptoms from those due to relapse.	Any spontaneously reported ‘new symptom’	16/51 (31.4%)
Yasui-Furukori et al. (2016)	DESS	3 or more DESS symptoms	14/25 (56%)

References for articles not cited in-text can be found in the supplementary material.

Abbreviations: AE, adverse event; DESS, Discontinuation-Emergent Signs and Symptoms.

a The figures used by Henssler et al. for these studies were based on any withdrawal-related symptom (criteria for the incidence analysis) measured by a structured instrument or specific questions relevant to antidepressant withdrawal.

**Figure 1.**
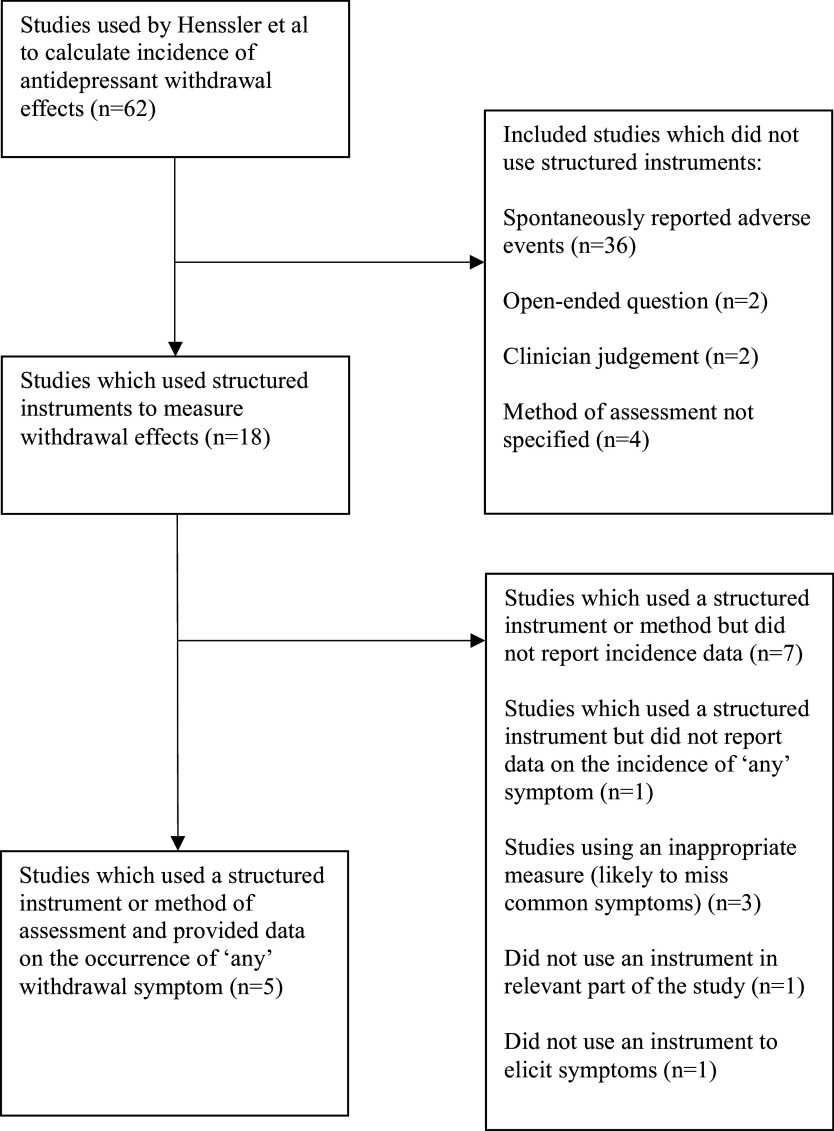
Flow diagram of studies included in Henssler et al.’s incidence calculation that used a structured instrument or method of assessment of antidepressant withdrawal symptoms.

Therefore, only 8.1% (5/62) of the studies included in Henssler et al.’s meta-analysis, involving 4.8% (601/12,603) of total participants, presented data derived from a systematic and relevant assessment of withdrawal symptoms (Figure 1).

## Discrepancies and uncertainties

Minor discrepancies in data extraction are common in systematic reviews, but some of those in the Henssler et al.’s review are likely to have impacted the results of the analysis, given the size of the studies involved (see Table 1).

For example, participants in a large, pooled analysis of studies of desvenlafaxine by Montgomery et al. (2009) were double counted, so that 947 participants from these studies were included in the meta-analysis twice.

Figures for several further studies are unreliable due to minimal reporting of adverse events, leading to uncertainty about the total number of people who were followed up after discontinuing their antidepressant (details in Table 1 and Supplementary Table S1). Henssler et al.’s use of the number randomized as the denominator in these cases would tend to reduce the rate of reported withdrawal effects, unless there were no dropouts (which is unlikely).

In two studies, all or some participants only reduced the dose of their antidepressant and did not stop (Bakish et al., 2014; Murata et al., 2010). The reductions made may not have had a large enough impact on receptor occupancy to trigger a withdrawal reaction (Horowitz & Taylor, 2019).

The study by Rapoport et al. should not have been included because the number of participants who experienced a withdrawal symptom is not reported. Henssler et al. used the number of people who withdrew from the trial due to a discontinuation-emergent adverse effect (Rapaport et al., 2001).

## Observation periods

Observation periods in the studies included in Henssler et al.’s review were generally short – the mode was two weeks. Short follow-up periods are likely to miss some withdrawal effects, which may not necessarily start immediately (Stockmann, Odegbaro, Timimi, & Moncrieff, 2018) due to receptor occupancy taking weeks to fall for many drugs (not just fluoxetine)(Sørensen, Ruhé, & Munkholm, 2022b) or the accumulation of downstream effects that are not well understood (Horowitz & Taylor, 2024).

## Duration of treatment

Previous research has shown that the incidence and severity of antidepressant withdrawal effects are greater following long-term use (Horowitz et al., 2023; NICE, 2022a).

The weighted average duration of exposure to antidepressants in the 58 studies included in the incidence analysis, which reported this data, was less than six months (23.4 weeks). In 30 of these, participants had used antidepressants for less than three months, and only nine involved a majority of participants who had taken antidepressants for a year or more (Table 1). Moreover, in two of these, figures that underestimate withdrawal events were inadvertently used in Henssler et al.’s analysis (Rapaport et al., 2001; Tourian, Pitrosky, Padmanabhan, & Rosas, 2011).

## Placebo withdrawal

Henssler’s final estimates were computed by subtracting the incidence of withdrawal effects reported following the discontinuation of a placebo (nocebo effects), in trials that reported this data, from the incidence rate among people who had withdrawn from an antidepressant, derived from a larger group of studies. Although the occurrence of nocebo effects, or the misclassification of non-specific symptoms as withdrawal-related effects, is an important consideration, the use of different groups of studies to estimate antidepressant and placebo withdrawal contravenes recommendations because of likely differences between the groups (Glenny et al., 2005).

Henssler et al.’s strategy also assumes that the adverse effects reported by people withdrawing from a placebo and an antidepressant are the same. However, it is unlikely that these are ‘like for like’.

Antidepressant withdrawal is associated with common, nonspecific symptoms such as dizziness, headache, and anxiety (as well as more specific symptoms, such as electric ‘zaps’). These will occur to some extent as ‘background noise’ in the placebo group, as highlighted by Baldwin, Montgomery, Nil, and Lader (2007). However, antidepressant withdrawal symptoms are likely to be more severe and occur more frequently. It has been reported that people can become so dizzy they have physical accidents (Moncrieff et al., 2024) or be referred for neurological workups (Haddad, Devarajan, & Dursun, 2001), for example. Therefore, incidental or background symptoms can only be distinguished from genuine withdrawal symptoms by measuring their severity and frequency, in the same way that symptoms of anxiety and depression are usually rated. Only one study included in Henssler et al. reported the severity of individual symptoms, but there was no placebo group in this study (Khan et al., 2014).

This point is supported by the fact that although withdrawal symptoms in general were only about twice as frequent among people taking an antidepressant compared to those taking a placebo in Henssler et al.’s analysis, the limited indicators of severe withdrawal used (see below) were almost five times more common in antidepressant users.

## Severity

Table 3 shows the data used by Henssler et al. to calculate the proportion of people experiencing severe withdrawal effects. This was not presented in the paper, and readers might assume the figures referred to withdrawal symptoms whose severity had been measured using an instrument, or at least to adverse effects that had been judged to be severe. However, in 11 of the 19 studies, the analysis was based on figures for adverse events that led to study discontinuation or on Serious Adverse Events (SAEs). The basis for the selection of these particular studies is unclear since others presented such data.

**Table 3.** Studies used in Henssler et al.’s analysis of the incidence of severe withdrawal (eFigure 2 in Henssler’s supplementary appendix)

Study	Definition of severe withdrawal used by Henssler et al.	Number of participants with ‘severe’ withdrawal/total number according to this definition
Dallal and Chouinard (1998)	Number of participants described by study authors as having ‘severe’ symptoms	6/8
Davidson (2001)	Number with discontinuation-emergent adverse event of dizziness rated as severe	1/50
Durgham (2019)	Number who discontinued due to a discontinuation-emergent adverse event	2/159
GlaxoSmithKline (1992)	Number with an SAE during or after antidepressant treatment^a^	2/202
Khan et al. (2014)	Number who discontinued due to ‘withdrawal symptoms’	5/285
Kragh-Sorensen et al. (1974)	Number derived from the statement ‘No withdrawal symptoms were observed. However, in two patients, mild headaches on both the second and third days were reported.’	0/10
Kramer et al. (1961)	Number described by the study authors as having ‘marked symptoms’	10/25
Markowitz (2000)	Number having an adverse event leading to discontinuation	0/72
Murata et al. (2010)	Number described by the study authors as having ‘very distressing symptoms’^b^	5/56
Perahia (2009)	Number who discontinued due to an adverse event in the placebo group at any time after randomization in the relapse prevention (maintenance) trial	3/142
Perahia et al. (2005) acute	Number who discontinued due to a discontinuation emergent adverse event	15/490
Perahia et al. (2005) acute extension	Number who discontinued due to dizziness	1/242
Rickels et al. (1993)	The number experiencing ‘moderate or marked withdrawal’, defined by study authors as an increase of 20 points or more on a benzodiazepine withdrawal checklist. The criteria were derived from a study comparing withdrawal from alprazolam, imipramine, and placebo.	0/11
Rosenbaum (1998)	Number who discontinued due to a discontinuation emergent adverse event	3/152
Rosenthal et al. (2013)	Number experiencing an SAE	1/272
Saxe (2012)	Number experiencing an SAE	1/178
Stein (2012)	Authors’ conclusion that there were no withdrawal symptoms^c^	0/114
Vandel (2004)	Number of adverse events (not participants) rated as ‘severe’	7/143
Zajecka (1998a)	Number who discontinued due to a discontinuation emergent adverse event	2/96

References for articles not cited in-text can be found in the supplementary material.

Abbreviation: SAE, serious adverse event.

a The SAEs in this study are described as occurring either during treatment with the antidepressants or in the 14-day period after discontinuation, so they are not necessarily related to withdrawal.

b Participants did not necessarily stop the drug completely in this study, and there were high rates of use of concomitant medications, including benzodiazepines (see Supplementary Table S1).

c According to Henssler et al. (personal communication), this was based on the authors’ conclusions. In the paper, the authors justify this on the basis that there was no excess risk of early relapse in the placebo group during the relapse prevention trial, and that the mean number of DESS symptoms following discontinuation of agomelatine and switch to placebo at the end of the trial was similar to the mean among those who continued agomelatine.

In any case, neither is a valid indicator of the severity of withdrawal symptoms. Decisions to discontinue from a trial involve many considerations. Researchers usually make concerted efforts to retain participants so as not to lose data and power, thereby making it likely that only unusually severe events culminate in someone leaving a trial. SAEs are a formal category of events with a precise definition, which includes events that lead to death, are life-threatening, lead to hospital admission, cause persistent or significant disability or incapacity, or a congenital abnormality (Health Research Authority, 2024). Therefore, there is a high threshold for categorizing an event as an SAE, and severe symptoms, even if painful, uncomfortable or debilitating, would rarely qualify, especially after short-term exposure.

The few studies that reported authors’ qualitative assessment of the severity of withdrawal symptoms yielded varied results. Some suggested that symptoms were generally mild (Kragh-Sorensen et al., 1974; Rickels, Schweizer, Weiss, & Zavodnick, 1993), and some suggested that they were commonly or not infrequently severe (Dallal & Chouinard, 1998; Kramer, Klein, & Fink, 1961; Murata et al., 2010). The authors of one noted that the symptoms of withdrawal in general were ‘fairly distressing and uncomfortable’ and that people who had severe withdrawal had ‘very distressing symptoms’ (p. 16) (Murata et al., 2010).

In another small study identified by Henssler et al. but not included in their analysis of severity, 12 of 14 participants who abruptly stopped fluvoxamine after 7–8 months experienced withdrawal symptoms, and of these five had to take time off work, six contacted researchers for help, three sought medical attention, one was re-medicated because of panic, and one became suicidal (Black, Wesner, & Gabel, 1993). Incidentally, it is also interesting to note that several studies documented rare cases of hospitalization and other serious events that were considered likely or possible complications of withdrawal (see Supplementary Table S1).

## Meta-analysis of studies using a systematic and relevant assessment of withdrawal symptoms

We identified five studies that conducted a systematic assessment of withdrawal symptoms using an appropriate structured instrument or method (Figure 1; Table 2). In all but one of these trials, participants had used antidepressants for 12 weeks or less. In one trial of paroxetine withdrawal, the mean duration of prior use was 106 weeks, but 59% of participants in this trial underwent a very slow withdrawal over a period of up to four years and not all participants discontinued their antidepressant. The majority were also using concomitant benzodiazepines and other drugs prescribed for depression and anxiety (Murata et al., 2010). All five studies were rated as having a low probability of the misclassification of withdrawal and relapse (Table 1).

**Figure 2.**
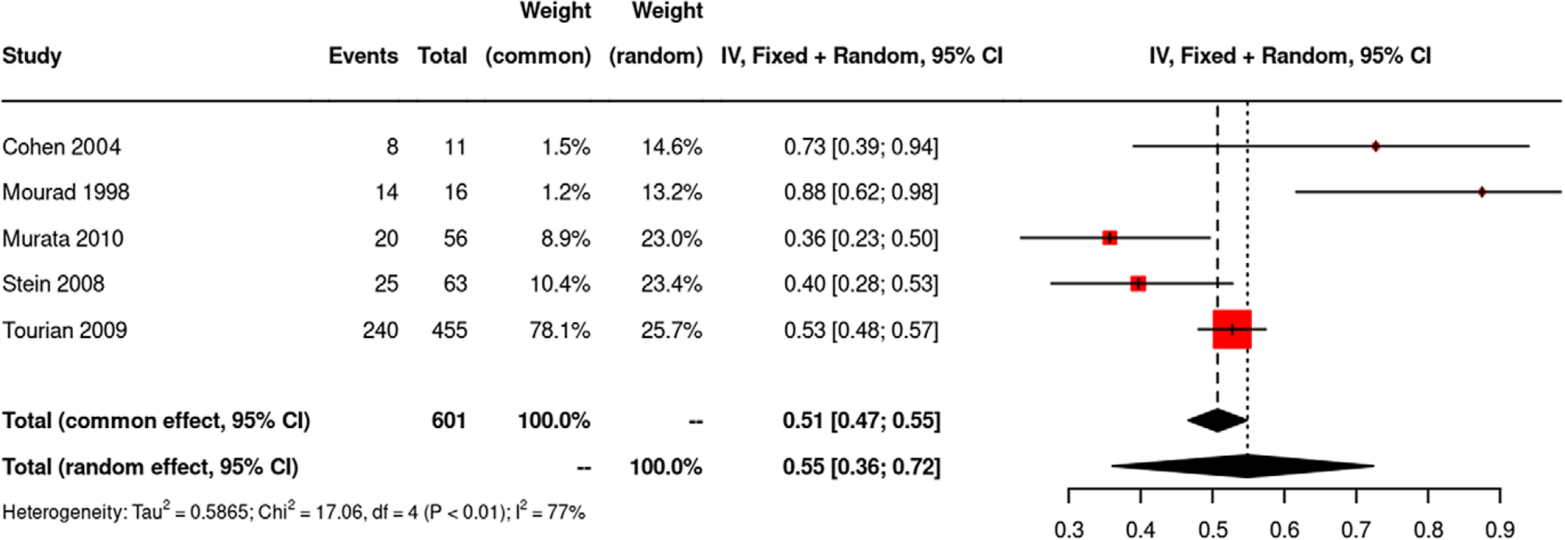
Meta-analysis of studies using a structured instrument or method of assessment of antidepressant withdrawal symptoms: Forest Plot.

The pooled rate of withdrawal symptoms in all five trials was 0.55 (95% confidence interval CI, 0.36–0.72, *N* = 601) using a random effects model, without subtracting nocebo effects (Figure 2). Heterogeneity was high (*I* ^2^ = 77%; *τ* ^2^ = 0.59; *Q* = 17.1, df = 4, *p* = 0.002). Excluding the trial by Murata, in which not all participants stopped their antidepressant, yielded a pooled estimate of 0.61 (CI 0.38–0.80; *N* = 545; *I* ^2^ = 74.4%; *τ* ^2^ = 0.67; *Q* = 11.7, df = 3, *p* = 0.009). Removing the trial of agomelatine (Stein, Ahokas, & de Bodinat, 2008), which has a different mechanism of action from other antidepressants and has consistently been found to have a low potential for dependence (Goodwin, Emsley, Rembry, & Rouillon, 2009; Montgomery et al., 2004), produced an estimate of 0.69 (0.43–0.87; *N* = 482; *I* ^2^ = 72.7%; *τ* ^2^ = 0.63; *Q* = 7.3, df = 2, *p* = 0.02) (see Supplementary material Figures S1–S2).

These figures are likely to include nocebo withdrawal or incidental symptoms. Although these were not reliably measured in the original review, for illustration, we deducted Henssler et al.’s estimate of placebo withdrawal in trials using a structured instrument (30%) from our estimates. This resulted in a range of 25%–39% of people experiencing withdrawal symptoms.

## Discussion

The data that form the basis of Henssler’s review were derived from trials, which were mostly funded by drug companies to assess efficacy, in which withdrawal was assessed cursorily, most often based on spontaneously reported adverse events. The problematic nature of such data is not discussed in Henssler’s paper, even though it is known to be inconsistent and unreliable and is particularly likely to miss emotional symptoms of withdrawal. This, and discrepancies in, and uncertainty of some of the figures, short duration of prior treatment, short observation periods, and other limitations, make most of the data unreliable and inadequate for the task of estimating the incidence of withdrawal. Likewise, the data selected for the analysis of severity were not justified or transparent and do not adequately represent the severity of withdrawal symptoms.

The limitations of the data may explain why there were no associations between the prevalence of withdrawal symptoms and pharmaceutical industry funding or length of prior antidepressant treatment across studies in Henssler et al.’s analyses. Differences between antidepressant agents and the relative lack of data from non-funded studies and studies with participants with longer durations of use may also have contributed to the failure to find differences.

Although nocebo or incidental withdrawal symptoms are relevant, Henssler et al.’s subtraction of the rate of placebo symptoms from antidepressant withdrawal symptoms is not justified. It does not account for the likely differences in the severity of symptoms following antidepressant and placebo withdrawal, and the estimates derive from different groups of studies.

Only five studies included in Henssler et al.’s meta-analysis of incidence had assessed withdrawal symptoms in a systematic and relevant manner. Depending on which studies were included, rates of withdrawal symptoms in these studies ranged between 55% and 69%, which reduced to between 25% and 39% after deducting Henssler et al.’s rate of nocebo withdrawal symptoms. However, since only one of these studies lasted longer than 12 weeks, these figures do not represent the effects of withdrawing from long-term treatment. They suggest withdrawal symptoms are common even after short-term use.

Conducting and obtaining funding for high-quality research on antidepressant withdrawal symptoms is challenging. Ideally, a randomized trial comparing people who are withdrawn from placebo or antidepressants after a clinically relevant duration of treatment is needed. Such a trial would need to employ a systematic and comprehensive measure of withdrawal symptoms, rated for frequency and severity, to have the best chance of distinguishing them from background events and symptoms of the underlying problem.

The results of Henssler et al.’s review have been interpreted as suggesting that antidepressant withdrawal is rare and unproblematic (Pariante, 2024), although we note this was not necessarily the conclusion of its authors. However, as we have shown, the review does not provide good grounds to make reliable judgments about withdrawal. Clinicians and patients need to be aware of its limitations to inform decisions about the use of antidepressants.

## Supporting information

Moncrieff et al. supplementary material Moncrieff et al. supplementary material

## Data Availability

All data associated with this manuscript have been published in the paper. Further enquiries can be directed to the corresponding author.

## Supplementary material

The supplementary material for this article can be found at http://doi.org/10.1017/S0033291725100652.
