# Supplementary material for: Evidence on antidepressant withdrawal: an appraisal and reanalysis of a recent systematic review
Source: Psychol Med. 2025 Jul 22;55:e191. doi: 10.1017/S0033291725100652 (PMC12315658; doi:10.1017/S0033291725100652)

**Supplementary Material**

**Supplementary Table 1 (Table S1): Further details on studies included in Henssler et al’s incidence analysis**

| Study | Measures (further data) | Discrepancies (further data) | Misclassification of withdrawal (further data) | Relevant events (e.g. SAEs) | Further information |
| --- | --- | --- | --- | --- | --- |
| Allgulander 2006 |  |  | Anxiety and withdrawal measured concurrently. Among the placebo group, 25/105 people relapsed within 14 days and the Kaplan Meier curve suggests a much larger proportion relapsed during the first 2 months and that this was when maximum divergence from the continuation arm occurred. It is also notable that anxiety was not documented as a withdrawal-related adverse event, although 'nervousness' was. | One patient was hospitalized with six SAEs (worsening of insomnia, worsening of dyspnoea, worsening of anxiety, mouth dry, fatigue, and concentration impaired) 17 days after randomization to placebo |  |
| Bainum 2017 | Authors' symptom list omitted common withdrawal symptoms |  | No assessment of psychiatric symptoms |  |  |
| Bakish 2014 |  |  |  |  | The antidepressant was not discontinued but reduced by between 50 and 75% of the original dose |
| Baldwin 2006 |  |  |  |  |  |
| Bhuamik 1996 | Henssler et al's figures were based on the statement 'Withdrawal symptoms were reported in five out of 12 patients' |  |  |  |  |
| Black 1993 |  |  | All symptoms considered as withdrawal symptoms | 1 patient became suicidal. 5 took time off work, 6 contacted researchers with concerns, 3 sought medical assistance. 1 became suicidal. 1 re-instated drugs due to severe panic. |  |
| Bourgeois 1991 | Discontinuation was evaluated 'by collecting clinical signs and symptoms' for one week after discontinuation with no further details |  |  |  |  |
| Ceccherini-Nelli 1993 |  |  | Depression scores and withdrawal measured concurrently, but there was a decrease in HAM-D scores over the course of the study | 1 developed ventricular extrasystolic beats. 2 developed hypomania with no history of hypomania. 1 developed resting tremor of the jaw, tongue, upper extremities and rigidity for 4 days (after stopping amitriptyline), 1 developed nocturnal enuresis |  |
| Charney 1982 | Nurses’ ratings only included psychological symptoms and not physical symptoms of withdrawal |  | All symptoms considered as withdrawal | 1 patient experienced panic, mood swings, agitation, hypomania after stopping amitriptyline |  |
| Clauw 2013 |  |  | Recurrence of pain and withdrawal measured concurrently, with some evidence of increased pain in the discontinued group (higher rates of drop out mostly due to worsening pain). |  |  |
| Cohen 2004 |  |  | No psychiatric disorder |  |  |
| Coupland 1996 |  |  |  |  |  |
| Durgam 2019 |  |  | Relapse and depression measured concurrently with withdrawal. Relapse was more common in the placebo group and occurred earlier. MADRS scores increased by 5 points in the placebo group over the course of the trial. There were no emotional symptoms amongst reported AEs |  |  |
| Fava 1997 |  |  |  |  |  |
| Favaro 2001 | ‘Symptoms typical of SSRI-withdrawal' identified retrospectively. No other details |  |  |  |  |
| Feiger 1999 |  |  | Relapse and withdrawal measured concurrently. Relapses occurred earlier on placebo randomisation. There were no emotional symptoms amongst reported AEs (only AEs with incidence ≥ 10% were reported). |  |  |
| Ferguson 2012 |  | Denominator unclear because the taper was 'modified' in 50 patients, including 18 in whom it was 'omitted'. |  |  |  |
| Gallagher 2012 |  |  |  |  | Results are presented in many different ways in the original paper and some suggest higher rates than the figures used by Henssler. e.g. overall rate of AEs during the taper period was 196/384. Mean scores on the DESS for all groups in the taper phase were between 5 and 10, suggesting most participants experienced more than 1 DESS symptom. |
| GlaxoSmithKline 1992 |  |  |  |  |  |
| Higuchi 2016 |  | The number randomised was 354 but the number who entered the taper and follow-up period was 307 |  |  |  |
| Ivgy-May 2015 |  | Denominator unclear because the total number discontinuing treatment was not given. Henssler used the number randomised but it is likely to be lower due to dropouts and loss to follow-up (total number of dropouts is not given, but 20 participants taking esmirtazapine discontinued the trial early due to AEs). | No psychiatric disorder |  |  |
| Jain 2012 |  | Denominator unclear because the total number discontinuing treatment is not given. Henssler used the number randomised but it is likely to be lower due to dropouts and loss to follow-up (numbers not reported). |  |  |  |
| Kamijima 2005 |  | 40 participants had 'treatment-related' AEs in the placebo group and Henssler et al added two who dropped out due to AEs, but these may have been included in the 40 participants. | Although withdrawal symptoms were measured concurrently with panic attacks and anxiety, there was little change in HAM-A or panic attacks overall in the placebo group. However, this does not exclude the possibility that some participants may have had increases due to withdrawal. There was also a non-significantly higher relapse rate in the placebo group, shorter time to relapse and a higher rate of discontinuation due to lack of efficacy |  | 83 participants in the placebo group had an 'all causality' AE during the double-blind phase |
| Khan 2014 |  |  |  | One participant from the abrupt discontinuation group reported a suicide attempt and hypertension 2 days after the end of the double-blind period. Two participants (one from the abrupt and one from the tapered discontinuation group) reported suicidal ideation during the double-blind period. |  |
| Koran 2003 |  |  | Depression and withdrawal symptoms measured concurrently. MADRS scores increased by 9.4 points in the placebo group during the double-blind phase (compared to a decrease of 2.3 points in the continuation group). No emotional symptoms are mentioned as adverse effects suggesting that these were not measured or classified as symptoms of relapse. |  |  |
| Kornstein 2006 |  |  | Recurrence and depression measured concurrently with withdrawal. Although only two recurrences occurred within the 14-day window for withdrawal, time to recurrence in the placebo group was significantly shorter and reduction of MADRS scores greater. The most common AEs listed do not include any emotional symptoms, suggesting emotional symptoms may have been overlooked or classified as symptoms of recurrence. |  |  |
| Kragh-Sorenson 1974 | No data from the checklist were presented. Henssler's figures were based on the author's statement "No withdrawal symptoms were observed. However, in two patients mild headaches on both the second and third days were reported." |  | The authors state no 'immediate depressive symptoms' were noted in the week following abrupt withdrawal. However, there is no mention of other emotional symptoms (including anxiety) and relapses were monitored concurrently and were noted to have occurred |  |  |
| Kramer 1961 |  |  |  |  |  |
| Liebowitz 2009 |  | The total number who tapered and were followed up is not reported. Henssler used the number randomised but it is likely to be lower due to dropouts and loss to follow-up. 55 dropped out prematurely including 15 who 'failed to return'. Also, the taper period could be 'omitted or prolonged as medically indicated' |  |  |  |
| Mago 2013 |  |  |  |  |  |
| Mallya 1993 |  |  |  |  |  |
| Mease 2010 |  | Denominator unclear because the total number followed up after discontinuing treatment is not reported. Henssler use the number randomised but only 77 of the 122 randomised completed the OL extension phase, although participants could also be tapered after leaving the study early. |  |  |  |
| Montgomery 2009 (flexible) the figures given are actually for all 9 short term fixed and flexible dose studies |  |  |  |  |  |
| Montgomery 2009 (fixed) |  | Double counted. The authors note that relatively few participants from the flexible dose studies were eligible and therefore the denominator for the fixed dose studies is almost as high as for the pooled fixed and flexible dose studies |  |  |  |
| Montgomery 2005 |  |  | Relapse and withdrawal measured concurrently. 48/91 relapses in the placebo group occurred in the first 2 weeks after randomisation and substantial divergence between the placebo and continuation groups occurred by 28 days. There were no emotional symptoms amongst reported AEs except ‘nervousness’. |  |  |
| Montgomery 2013 |  | Denominator unclear because the total number followed up after discontinuing treatment is not given. Henssler use the number randomised, but only 225 participants in the milnacipran group (out of 278 randomised) completed the double-blind portion of the study prior to the taper phase. |  |  |  |
| Mourad 1998 |  |  | All symptoms considered symptoms of withdrawal |  |  |
| Murata 2010 |  |  | Withdrawal symptoms used in the incidence calculation measured using a scale similar to the DESS |  | Not all participants stopped their antidepressant, some only reduced the dose (no further details provided). There was a high rate of use of concomitant medication, with 71% participants using benzodiazepines, 75% tandospirone and 45% using 'nonbenzodiazepines' . |
| Oehrberg 1995 |  |  |  |  |  |
| Otani, 1991 | Henssler's figures are based on the statement: 'No withdrawal symptom was observed in any of these 21 patients. In the remaining 1, moderate headache was noted on the 14th day of dose reduction (from 30 to 20 mg).' |  |  |  |  |
| Perahia 2009 |  |  |  | One participant withdrawn from duloxetine experienced 3 SAEs: convulsion, hypertension and hypertensive crisis. Figures for adverse events in the double-blind phase following randomisation to placebo are also presented (89/142, 62.7%) but not used by Henssler et al. |  |
| Perahia 2005 acute studies |  |  |  |  |  |
| Perahia 2005 extension studies |  |  |  |  |  |
| Rapaport 2001 |  | Figures represent the number of participants randomised to placebo who subsequently withdrew from the trial due to an adverse event, not the number who experienced any adverse event, which is not reported. | Relapse, symptoms and withdrawal measured concurrently. Panic and anxiety scores increased to a greater degree in those randomised to placebo (though the difference was not statistically significant). Withdrawals due to lack of efficacy were higher among those randomised to placebo and the Kaplan Meier curve indicates early onset of acute exacerbation among the placebo group. There were no emotional symptoms amongst reported AEs except depression. |  |  |
| Raskin 2003 (results and details reported in Perahia, 2005) |  |  |  | 17.2% of the 793 events reported were rated as severe |  |
| Ravindran 2007 | Henssler's figures are based on the statement 'No patients reported any discontinuation symptoms on medication termination' |  | Low potential because participants do not have a psychiatric disorder |  |  |
| Rickels 2010 open label study |  | The total number who were followed up after discontinuing venlafaxine is unclear because the taper could be 'extended, shortened, or omitted at the discretion of the investigator' (no further details provided) |  |  |  |
| Rickels 2010 relapse prevention |  |  |  |  |  |
| Rosenthal 2013 |  | The total number assessed post discontinuation is not reported. It is likely to be lower than 300 (the number randomised plus those finishing OL treatment and not entering the trial) due to dropouts and loss to follow-up. Only 210 participants completed the DB phase, including 10 lost to follow-up. |  |  |  |
| Santonastaso 2001 | Henssler's figures appear to be based on the following statement: 'Two patients reported withdrawal symptoms such as dizziness, irritability, and insomnia after sertraline discontinuation.' P 147 |  |  |  |  |
| Saxe 2012 |  |  | Ratings of poor mental health, fatigue and pain measured concurrently with withdrawal symptoms increased following discontinuation of milnacipran. These symptoms were considered as condition-related outcomes, despite all being reported (including pain) following SNRI withdrawal (Fava et al., 2018). There were no emotional symptoms amongst reported AEs following withdrawal | One participant randomised to milnacipran discontinuation experienced hyponatremia, a grand mal convulsion and a confusional state and was withdrawn from the study. |  |
| Stein 1996 |  | How withdrawal symptoms were assessed is not specified and Henssler's figures seem to be based on the following statement: 'In retrospect, it seems that two patients randomized to placebo were experiencing withdrawal symptoms (i.e., dizziness and nausea) at the time of relapse'. | Relapse and withdrawal measured concurrently. Five of the 8 patients on placebo relapsed. The withdrawal symptoms mentioned later in the article do not include any emotional symptoms. |  |  |
| Stein 2008 |  |  | Withdrawal symptoms used in the incidence calculation measured using the DESS |  |  |
| Steiner 2005 |  |  | Psychiatric symptoms were not measured |  |  |
| Tourian 2011 |  | The number of participants who discontinued the study drug and were followed up subsequently is unclear. The authors specified that only 1,249 patients were eligible to enter the taper period (144 withdrew early and 2 had no record of taper). Of the 1249, only '678 patients tapered per protocol, 146 patients had their taper period extended, 74 had their taper period shortened, and 351 had their taper period omitted at the discretion of the investigator.’ It is not clear whether 'omitted' means they stopped the study drug abruptly without tapering or whether they didn't stop (possibly continuing on a non-study drug). Taking those who were eligible for the taper period as the denominator, 584/1249 (46.8%) had a withdrawal-emergent adverse event. Taking the number who tapered per protocol or had the taper shortened, it was 584/752 (77.6%). |  | ‘Three patients...had SAEs considered possibly, probably, or definitely related to the study drug during the poststudy period (hyperthyroidism, 1 patient; convulsion, 2 patients)' |  |
| Tourian 2009 | 'Specific AEs were queried by the investigator' in the taper phase P 1408. |  | Specific withdrawal-related AEs used in the incidence calculation were elicited alongside the DESS |  |  |
| Tyrer 1984 |  |  | Relapse and withdrawal were measured concurrently. 12/51 participants were classified as relapsing and mean anxiety and depression rating scale scores also increased. Assessors worked on the assumption that only symptoms which increased then decreased in the observation period (4 weeks) were withdrawal effects whereas symptoms which persisted were relapse, however withdrawal symptoms are not necessarily short-lived. |  |  |
| Vandel 2004 |  |  |  |  |  |
| Wade 2007 |  |  |  |  |  |
| Yasui-Furukori 2016 |  |  | Psychiatric symptoms were not measured and withdrawal symptoms were measured comprehensively (thus low potential for misclassification) | Severe withdrawal occurred in 2/25 patients defined as ‘requiring reinstatement’ | A relatively long taper was employed of 20 weeks. |
| Zajecka 1998a |  | Non-optimal figures (adverse events at 6 weeks). Adverse events during the course of the 6 weeks were 64/96 in the placebo (discontinued) group | Since this was part of a trial of maintenance treatment, relapse and symptoms would have been measured concurrently with withdrawal symptoms, but they were not reported in the paper |  |  |
| Zajecka 1998b (conference abstract) |  |  | Relapse and withdrawal were measured concurrently, but no data is presented |  | At 28 days post randomisation 38 of the 130 participants in the placebo group reported at least one 'new onset adverse event'. |

OL open label; AE adverse event; SAE serious adverse event; HAM-A Hamilton Anxiety Rating Scale; MADRS Montgomery-Asberg Depression Rating Scale; DESS Discontinuation-Emergent Signs and Symptoms

**References**

Fava, G. A., Benasi, G., Lucente, M., Offidani, E., Cosci, F., & Guidi, J. (2018). Withdrawal Symptoms after Serotonin-Noradrenaline Reuptake Inhibitor Discontinuation: Systematic Review. *Psychotherapy and Psychosomatics*, *87*(4), 195–203. doi: 10.1159/000491524

**Henssler et al (2024) re-analysis: analysis plan**

**Introduction**

Antidepressant use, including long-term use, is common and rising (Burns et al, 2022; Kendrick, 2021). It is accepted that many antidepressants provoke a withdrawal syndrome when they are stopped, but the frequency and clinical significance of this has been debated. We intend to appraise and re-analyse the evidence presented in a recent systematic review by Henssler et al (2024). We will focus on studies that use a systematic method of assessment of withdrawal symptoms, such as the DESS or a similar structured instrument or method.

**Research question**

What is the incidence of antidepressant withdrawal in studies included in the recent Henssler et al (2024) systematic review and meta-analysis that use a structured instrument or method to assess withdrawal? We will use the same definition of withdrawal as Henssler et al, namely the occurrence of at least one withdrawal symptom.

**Study criteria**

***Inclusion criteria***

In line with Henssler et al, we will include randomized controlled trials, cohort-studies, case-control-studies and descriptive cross-sectional studies. However, for our meta-analysis, we will include only studies that employ a systematic method, such as a structured instrument, for identifying and measuring withdrawal symptoms.

***Exclusion criteria***

Studies that only include spontaneous or unspecified reporting of withdrawal effects.

**Primary outcomes**

We will use the same outcome and definition as Henssler et al (2024). Thus we will assess the proportion of people experiencing at least one symptom of antidepressant withdrawal.

**Secondary outcomes**

We will not look at secondary outcomes

**Statistical analyses**

As in the original review, we will analyse the proportion of participants who report at least one withdrawal symptom and we will conduct the meta-analysis of proportions using the Logit method based on the inverse variance. We will use R’s “meta” package to conduct the analysis. Heterogeneity will be explored by inspection of Forest plots and computation of the I^2^ statistic. We will conduct a sensitivity analysis excluding studies using agomelatine due to it being less likely to provoke withdrawal effects.

24^th^ July 2024

Figure S1 Studies using structured instruments or methods of assessment excluding Murata 2010


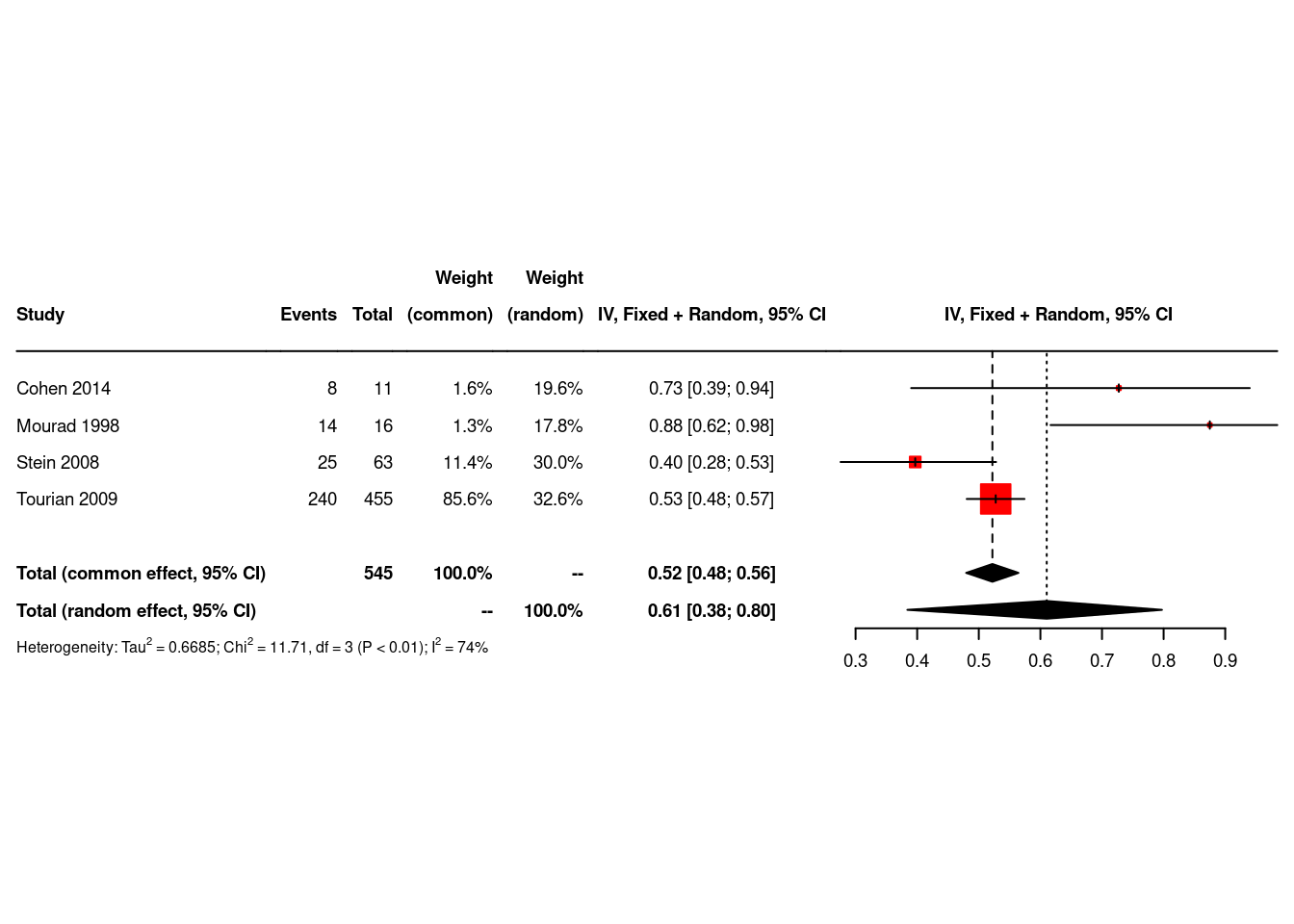


Figure S2 Studies using structured instruments or methods of assessment excluding Murata 2010 and Stein 2008


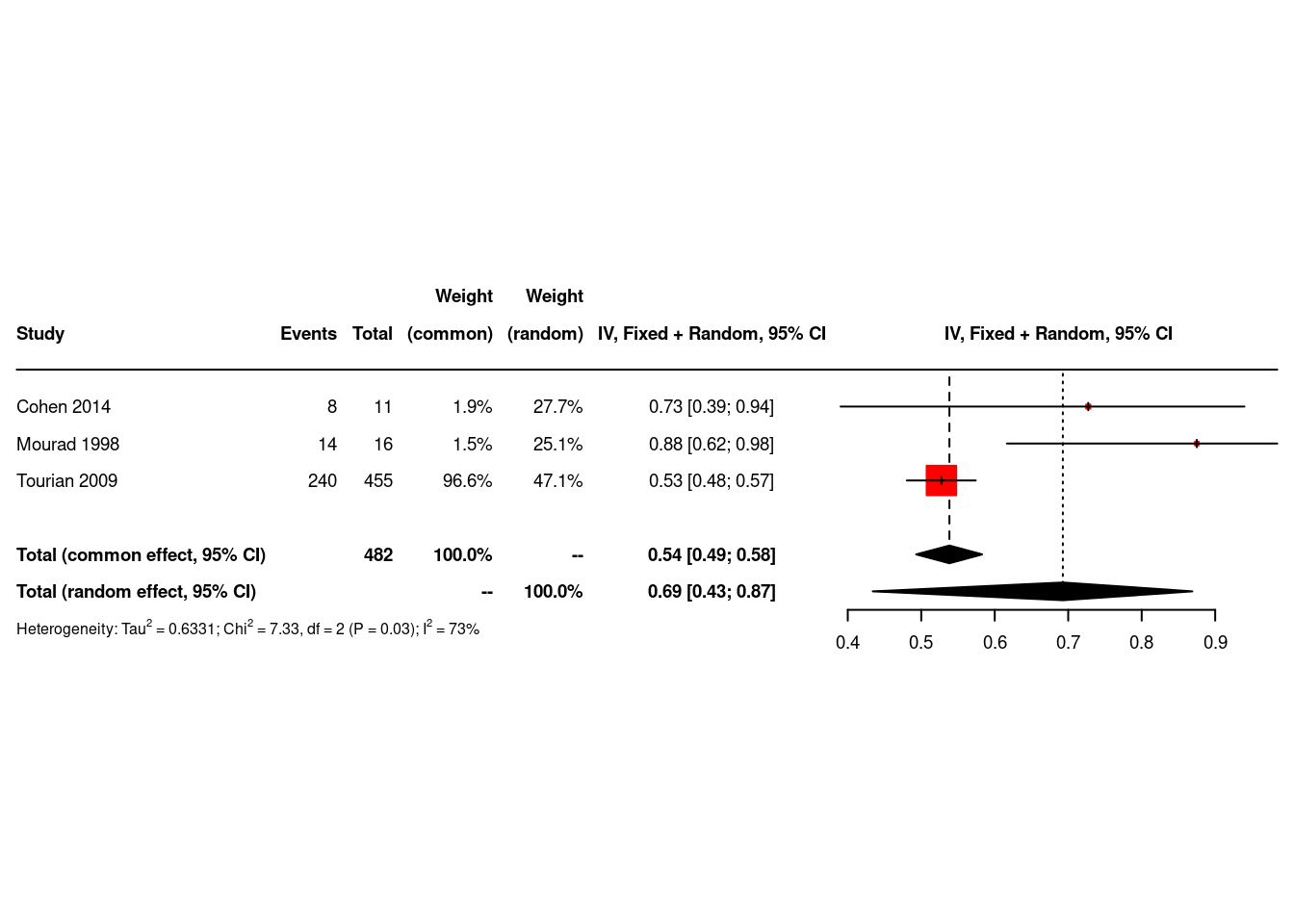

Supplement: Moncrieff et al. supplementary material [file S0033291725100652sup001.docx]
